# Set-Based Discriminative Measure for Electrocardiogram Beat Classification

**DOI:** 10.3390/s17020234

**Published:** 2017-01-25

**Authors:** Wei Li, Jianqing Li, Qin Qin

**Affiliations:** 1 School of Instrument Science and Engineering, Southeast University, 2 Sipailou, Nanjing 210096, China; ljq@seu.edu.cn (J.L.); qq_nj@seu.edu.cn (Q.Q.); 2 School of Basic Medical Sciences, Nanjing Medical University, 101 Longmian Avenue, Nanjing 211166, China

**Keywords:** ECG beat classification, set-based discriminative measure, metric space, set-based dissimilarity

## Abstract

Computer aided diagnosis systems can help to reduce the high mortality rate among cardiac patients. Automatical classification of electrocardiogram (ECG) beats plays an important role in such systems, but this issue is challenging because of the complexities of ECG signals. In literature, feature designing has been broadly-studied. However, such methodology is inevitably limited by the heuristics of hand-crafting process and the challenge of signals themselves. To address it, we treat the problem of ECG beat classification from the metric and measurement perspective. We propose a novel approach, named “Set-Based Discriminative Measure”, which first learns a discriminative metric space to ensure that intra-class distances are smaller than inter-class distances for ECG features in a global way, and then measures a new set-based dissimilarity in such learned space to cope with the local variation of samples. Experimental results have demonstrated the advantage of this approach in terms of effectiveness, robustness, and flexibility based on ECG beats from the MIT-BIH Arrhythmia Database.

## 1. Introduction

As stated by the World Health Organization, cardiovascular diseases are the primary cause of death worldwide. To diagnose heart diseases, electrocardiogram (ECG) signal analysis is one of the most commonly used tools at the early stage. ECG signals record the cardiac electrical activity, and can provide important pathological information about human cardiac condition. However, it is actually impractical for doctors to analyze large amounts of ECG records in a short period of time, due to the limited ability of human eyes as well as the complicated variation of ECG signals themselves. Hence, the Computer Aided Diagnostic (CAD) system has attracted growing attention in recent years [1,2].

As a non-invasive method for ECG signal analysis by CAD, heartbeat classification is important to recognize the heart arrhythmias. The ECG beats usually suffer from the changing amplitude and duration of waveforms caused by the real-scenario noises and the signal chaotic nature, which dramatically increase the challenge to decipher the hidden beat type information contained within the data.

In literature, methodologies for ECG beat classification can be summarized into two stages. The first stage is feature extraction. This stage transforms the raw signal into the meaningful discriminatory quantities. The second stage is classification. This stage distinguishes the sample classes by pattern recognition and/or machine learning techniques.

Feature extraction plays an important role in the method pipeline because this stage establishes a platform for the subsequent procedures of pattern recognition and/or machine learning. Technologies to extract ECG signal features have been studied and developed from different angles, such as description of waveform morphologies, representation of waveband statistics, quantization of wavelet coefficients, and so forth [3,4,5,6,7,8,9,10,11,12,13,14,15].

ECG signals show sophisticated variations for different patients across temporal and physical conditions. Even for the healthy subject, the heartbeats will not be the same from one to the other. Although feature methodology tries to quantify ECG in a discriminative way, the heuristics of hand-crafting process and the complicated variation of signals unavoidably impede the performance enhancement. Therefore, pattern analysis and/or machine learning techniques play an important role in further improving the classification performance based on the extracted features [16,17,18,19,20,21,22].

To our knowledge, the main problem haunting this issue is large intra-class variation and small inter-class difference, which is caused by the facts that the same ECG beat type may present different characteristics across subjects and conditions, but different beat types may exhibit similar morphological and statistical properties. Furthermore, because ECG data suffer from variations, they are usually not independent and identically distributed between training and testing domains, which can easily lead to the over-fitting problem. On the other hand, for multi-class classification, the challenge increases with the number of classes, especially when the data undergo severe variations. Although the Association for the Advancement of Medical Instrumentation (AAMI) standards group all ECG beats in the MIT-BIH Arrhythmia Database into five classes [4,5,7,9,11,23,24,25,26,27,28], in diagnosis, the finer classification on 16 detailed beat types will provide more valuable information. However, in such cases, the increased class number will unavoidably complicate the classification process.

To address these problems, we propose a novel approach, named “Set-Based Discriminative Measure”. This approach recasts the issue of ECG beat classification into the problem of set-based dissimilarity measure. More specifically, we first learn a discriminative metric space using the training data to pull the same-class testing samples close together whilst pushing the different-class ones far apart; then, we measure the new dissimilarities between the query set in the testing data domain and the corpus sets in the training data domain. Based on these dissimilarity scores, we can rank the corpus sets and match the query set in question to the top-rank class. In practice, the ECG signals actually belong to time-series data. In most cases, the specific arrhythmia heartbeats tend to occur frequently and contiguously instead of only once for the patients if they suffer from some cardiovascular diseases. It is not difficult to obtain the beat data set by means of automatic analysis or human-computer interaction. Moreover, the large set for the fixed beat type can also be collected by combining the subsets under different circumstances based on the existing pattern re-identification/matching techniques.

To sum up, there are three novel aspects in our approach. Firstly, we design a new scheme to classify the ECG beats. Different than the previous scheme pipeline “feature + classifier” that directly outputs the final decision, our proposed method solves the problem in the mode “feature + metric + measure” from the dissimilarity measure perspective. Measuring dissimilarity is convenient for generating the ranking results in the specified order, and the ranking sequence has an advantage in tolerating the correct match to appear in the top several positions, especially when the target beat types are ambiguous to distinguish. Secondly, we suggest measuring the set-based dissimilarity instead of sample-based distance. Traditional ECG beat classification focuses on the single sample manner. By contrast, the set contains the distribution information in the local area, which provides more opportunities for the dissimilarity measure to work against the potential abnormal behavior of the single sample. To better exploit the within-set information, we design a new set-based dissimilarity in our model. Last but not least, we employ the metric learning approach for projecting ECG beat features into a more discriminative space. Metric learning warps the feature space to satisfy the relative comparison relationship between intra-class distances and inter-class distances, which directly copes with the main problem of large intra-class but small inter-class distances in ECG beat classification.

## 2. Materials

We use MIT-BIH Arrhythmia Database for tests [29]. This benchmark database has been provided systematically for research studies on ECG analysis and can be obtained from the Physionet website [30]. The source of ECG data in this database was the collection of 4000 long-term Holter recordings taken from the arrhythmia laboratory from 1975 to 1979.

In total, this database contains 48 recordings, which were selected to comprise the normal beat and common types of life threatening arrhythmias. For these recordings, the subjects were 25 men aged from 32 to 89 and 22 women aged from 23 to 89. The first 23 recordings (numbered from 100 to 124 with some numbers missing) include the representative samples that arrhythmia detectors may encounter during routine clinical use. The remaining 25 recordings (numbered from 200 to 234, again with some numbers missing) cover a variety of rare but clinically important phenomena that might present significant difficulty to arrhythmia detectors. There are two channels of signals for each recording. The first channel is modified limb lead II in most cases, and occasionally modified lead V5. The second channel is usually modified lead V1, and sometimes V2, V4 or V5. All the signals last slightly longer than 30 minutes. These signals have been processed by a band-pass filter at 0.1–100 Hz and sampled at 360 Hz. We use the data from both channels for experiments. For convenience, we denote the dataset based on the first channel as MLII, and that based on the second as MLV.

Because each recording in MIT-BIH Arrhythmia Database is continuous in nature, it is necessary to extract the single heart beat from the signals for further processing. Actually, this database also has provided annotations for both beat class information and timing information verified by the independent experts. Since current capable R-peak detection algorithms have achieved more than 99% positive predictive accuracy and sensitivity [31,32,33], we directly use the prepared R-peak annotation file. Based on the R-peak position identified in the annotation file, we extract 235-point segments at R peaks from the recordings. For each segment, there are 90 sampling points before the R peak and 144 sampling points after it. If sampling points are inadequate before the first or after the last detected QRS complex in any of the signal files, then the corresponding beat is neglected. Finally, we acquire more than 109,000 beats from 16 different beat types altogether. Note that there are no universal standards for restricting the length of beat segments. Longer or shorter beats segments are also acceptable for experiments, and they do not affect the method demonstration. Thus, we don’t plan to look into this aspect in the paper.

## 3. Methods

In brief, there are three components in the proposed model: feature representation, metric learning, and set-based dissimilarity. Feature representation converts original ECG beats into the meaningful measurable quantities, which thus provides a platform for the subsequent processing. Metric learning optimizes a discriminative metric to improve the feature space by compacting the same-class samples whilst separating the different-class samples as far as possible. In the learned metric space, set-based dissimilarity measures the distance between query and corpus sets, and the classification/ranking results can be determined by these distance scores. We will launch detailed elaboration on each model component in this section.

### 3.1. Feature Representation

As the first component, feature representation plays a fundamental role in ECG classification. Every year, many new features emerge and achieve encouraging results in literature. In consideration of the latest research progress in wavelet transform based features [6,24,34,35,36,37,38], we represent the ECG data by this family.

More specifically, we mainly use wavelet Bi-orthogonal 6.8 (Bior 6.8) for experimental demonstration. Bior 6.8 decomposes the signals successively up to eight levels that include the low frequency and high frequency components. The low frequency component is called “approximation”, and the high frequency component is called “detail”. After decomposition, we calculate the maximum, minimum, mean, and variance of the coefficients from all the detail sub-bands and approximation sub-band, and then concatenate these statistical values into one 36-dimensional feature vector. Note that this feature has implicitly handled the baseline wander problem during quantification and representation, so we don’t have to do the extra data pre-processing of baseline wander elimination.

It is worth mentioning that, besides Bior 6.8, we also adopt several different wavelets to construct feature spaces for experimentation. Honestly, the value of the proposal lies in the incremental performance from the basic feature space. Although feature representation is not the focus of this paper, we admit that introducing a better feature can lead to an improved result, just like standing on the shoulders of giants.

### 3.2. Metric Learning

The purpose of feature representation is to construct a discriminative space in which sample classes can be easily distinguished. However, because of the complexity of ECG data, the phenomenon of class inseparability still exists even in a carefully-designed feature space, which more or less affects the classification results. Therefore, we resort to the Mahalanobis metric learning approach to transform the structure of feature space, in pursuit of the goal that intra-class distances are smaller than inter-class distances for all samples. To this end, we interpret multi-class classification from the viewpoint of ranking order, and employ the Metric Learning to Rank (MLR) model [39]. MLR directly optimizes the list-wise ranking, for which only the rank of the first correct match is counted, while the ranks of both other correct matches and any incorrect matches are arbitrary. Thus, optimizing ranking is consistent with the multi-class situation during ECG beat classification: there are large amounts of ranking instantiations of a given ground truth. MLR is described in the followings.

Given query collection $Q=\left\{q|q\in R^{d}\right\}$, which consists of query sample vectors, and corpus collection $X=\left\{x_{i}|x_{i}\in R^{d}\right\}$, which consists of corpus sample vectors, we suppose that *w* is the Mahalanobis metric matrix intended to optimize, and $\phi_{qi}\left(q,x_{i}\right)$ is a kind of matric representation of a corpus sample $x_{i}$ with regard to *q*:

(1) $$\begin{array}{c} \phi_{qi}\left(q,x_{i}\right)=-\left(q-x_{i}\right){\left(q-x_{i}\right)}^{T}. \end{array}$$

A desired ranking model can be defined by

(2) $$\begin{array}{c} g_{w}\left(q,x_{i}\right)=w^{{}^{T}}\phi_{qi}\left(q,x_{i}\right) \end{array}$$

for scoring $x_{i}$, and the ranking can be done by sorting scores in a descending order.

To learn *w*, usually, a joint feature map is adopted to represent the whole set of ranked data X. Let $y_{q}^{\mathrm{ranking}}\in Y$ be a ranking of X with respect to *q*, and $\psi \left(q,y_{q}^{\mathrm{ranking}},X\right)\in R^{d}$ be a vector-valued joint feature map, which is defined as the partial order feature:

(3) $$\begin{array}{c} \psi \left(q,y_{q}^{\mathrm{ranking}},X\right)=\sum_{x_{i}\in \chi_{q}^{+}}\sum_{x_{j}\in \chi_{q}^{-}}\left(\frac{\phi_{qi}\left(q,x_{i}\right)-\phi_{qj}\left(q,x_{j}\right)}{|\chi_{q}^{+}\left|\right|\chi_{q}^{-}|}\right), \end{array}$$

where $X_{q}^{+}$ denotes the set of relevant corpus samples with regard to the query sample *q*; $X_{q}^{-}$ denotes the set of irrelevant corpus samples with regard to the query sample *q*; and |•| means the set cardinality.

One important property of $\psi (q,y_{q}^{\mathrm{ranking}},X)$ is that, for a fixed *w*, the ranking $y_{q}^{\mathrm{ranking}}$ which maximizes $w^{T}\psi \left(q,y_{q}^{\mathrm{ranking}},X\right)$ can be obtained by sorting $g_{w}\left(q,x_{i}\right)$ in order of the descending scores.

The best *w* is expected to be the one that simultaneously makes

(4) $$\begin{array}{c} y_{q}^{*}=\arg\max_{y_{q}^{\mathrm{ranking}}}w^{T}\psi \left(q,y_{q}^{\mathrm{ranking}},X\right), \end{array}$$

where $y_{q}^{*}$ is the ground truth ranking of X for *q*. Thus, *w* can be learned by solving the following optimization problem:

(5) $$\begin{array}{c} \arg\min_{w}\mathrm{tr}\left(w\right)+\frac{C}{\left|Q\right|}\sum_{q}\xi_{q} \end{array}$$

s.t.

$$\begin{array}{c} \begin{array}{c} \begin{array}{c} w^{T}\psi \left(q,y_{q}^{*},X\right)\geq w^{T}\psi \left(q,y,X\right)+\Delta \left(y_{q}^{*},y\right)-\xi_{q},\\ \forall q,y\neq y_{q}^{*}; \end{array}\\ \xi_{q}\geq 0,\forall q, \end{array} \end{array}$$

where $y_{q}^{*}$ is the ground truth ranking of X for a given q∈Q, and $\xi_{q}$ is the slack variable, *C* is the trade-off parameter balancing between the slack variable and the regularizer, and $\Delta (y_{q}^{*},y_{q}^{\mathrm{ranking}})$ is the loss function to penalize predicting $y_{q}^{\mathrm{ranking}}$ instead of $y_{q}^{*}$, defined by $\Delta \left(y_{q}^{*},y_{q}^{\mathrm{ranking}}\right)=1-S\left(q,y_{q}^{\mathrm{ranking}}\right)$, in which

(6) $$\begin{array}{ccc} S_{\left(q,y\right)} & = & \frac{1}{\left|Q\right|}\sum_{q\in Q}\left\{\begin{array}{cc} 1/r_{q}, & r_{q}\leq k,\\ 0, & r_{q}>k, \end{array}\right. \end{array}$$

where $r_{q}$ is the position of the first relevant item in response to *q* in *y*; and *k* is the number of top ranked items to be considered. If the position of the first relevant item in response to *q* in *y* is larger than *k*, the reciprocal rank score will be penalized to be 0. Here, we just directly follow the commonly-used setting k=3 in the relevant works by default, which in some sense considers the balance of avoiding the penalty from being either over strict or over loose [40,41,42,43,44]. Additionally, in Problem 5, the $l_{2}$-norm based regularizer ${∥w∥}^{2}/2$ is also an alternative choice [39].

For the issue of ECG beat classification, the distribution gap between unknown testing data and prepared training data is ubiquitous, which aggravates the over-fitting problem. Actually, MLR has an advantage in tackling such an over-fitting problem, which is also one reason for us to employ this model [44]. MLR is based on the structural Support Vector Machine (SVM) framework. For machine learning approaches which directly work on Empirical Risk Minimization (ERM), minimizing the empirical risk cannot be guaranteed to be equivalent to minimizing the expected risk when the number of training samples is limited and the dimensionality is high, so it is easy for such kinds of models to incur over-fitting. SVMs have an advantage in avoiding over-fitting because SVMs use the spirit of the Structural Risk Minimization (SRM) principle. The SRM principle addresses over-fitting by balancing the model’s complexity against its success at fitting the training data. SVM learning actually minimizes both the Vapnik–Chervonenkis dimension and the approximation error at the same time. MLR is a structural SVM model, so it also inherits the robust ability against over-fitting. Hence, the inherent nature of MLR is conducive to alleviating the stubborn over-fitting problem during the learning process.

Because measuring Mahalanobis distance with the learned metric is equivalent to measuring Euclidean distance in the space projected by the decomposed metric *l* where $w=l^{T}l\to l=w^{1/2}$, in order to favor the set-to-set dissimilarity measure in the next step, we take advantage of such *l* to project the feature space.

### 3.3. Set-Based Dissimilarity

Although the projected space has been improved by the learned metric globally, due to the challenges of real ECG data, there still exists a portion of samples with unexpected deviation in the local areas. To reduce the impact from the irregularly-distributed samples, we recommend measuring the set-to-set dissimilarity instead of traditional point-to-point distance that merely focuses on the isolated two samples. Set-based dissimilarity is not vulnerable to the irregular behavior of individual samples if it suitably makes use of the within-set resource during measurement.

There are many kinds of set-based dissimilarities in a variety of applications, such as Minimum Point-wise Distance (MPD), Average Point-wise Distance (APD), Mean Approach Distance (MAD), and so on [42]. Inspired by the success of traditional dissimilarities for the classification topics in computer vision fields, we design a new dissimilarity: “Minority-Based Dissimilarity (MBD)”. This dissimilarity takes within-set variability into account by measuring the closest local minorities of each paired sets, with the assumption that the outer samples of sets are more informative for discrimination than the central parts.

Let us denote *a* an arbitrary point in set *A*, *b* an arbitrary point in set *B*, *l* the point-to-point distance, and *d* the point-to-set distance. Thus, MBD between two sets is given by:

(7) $$\begin{array}{ccc} D^{\mathrm{MBD}}\left(A,B\right) & = & \frac{1}{|A_{u}|}\sum_{a\in A_{u}}d(a,B)⊙\frac{1}{|B_{v}|}\sum_{b\in B_{v}}d(A,b), \end{array}$$

where the abstract symbol ⊙ can be defined as sum-operation, max-operation, or min-operation; by default, we make this operation as the sum-operation in this paper; $A_{u}$ is the subset of *A* nearest to *B*, and $B_{v}$ is the subset of *B* nearest to *A*; d(a,B)=min{l(a,b)|b∈B}, and d(A,b)=min{l(a,b)|a∈A}; and |•| means the set cardinality.

In fact, MBD has a close relationship with MPD and MAD. MPD is formulated as $D^{\mathrm{MPD}}\left(A,B\right)=\min_{a\in A,b\in B}d\left(a,b\right)$; and MAD is formulated as $D^{\mathrm{MAD}}\left(A,B\right)=\frac{1}{\left|A\right|}\sum_{a\in A}d(a,B)+\frac{1}{\left|B\right|}\sum_{b\in B}d(A,b)$. Hence, MBD can be degenerated into MPD if $|A_{u}\left|=\right|B_{v}|=1$ when symbol ⊙ is defined as the min-operation, and into MAD if $|A_{u}\left|=|A\right|$ and $|B_{v}\left|=|B\right|$ when symbol ⊙ is defined as the sum-operation. Therefore, MPD and MAD can be treated as the special cases of MBD.

In summary, set-based dissimilarity and metric learning solve the variation problem of ECG features from different perspectives. Metric learning relies on optimizing the global space metric for all the samples, while set-based dissimilarity takes advantage of within-set information in the local area during measure. In addition, the reason for metric learning to complement set-based dissimilarity is that the behavior of closing the same-class samples and distancing the different-class samples can potentially improve the relative comparison relationship among sets with the target of better discrimination.

## 4. Results

### 4.1. Experimental Setup

**Figure 1:**
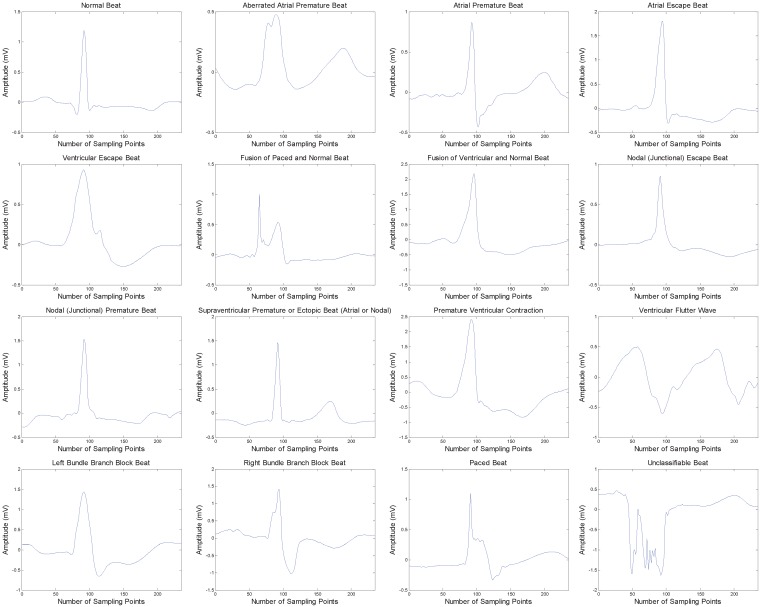
Illustration of 16 types of electrocardiogram (ECG) beats.

The details of 16 ECG beat types are given in Table 1. It can be seen from this table that data distribution among 16 classes is quite imbalanced. Class N has the largest size which is even more than 75,000, while the size of the smallest class S is only 2. In addition, other class sizes vary from tens to thousands. Such imbalance more or less enhances the classification difficulty. The beat data are collected from 235-point segments of the ECG recordings, so the original data dimension is 235. We illustrate these data in Figure 1 by sampling the beat from each class. The waveform display may not be exactly medically-standard because we directly use the real data which suffer from variations and noises. However, these waveforms just reflect the challenge we meet in this issue: ECG beat data are complicated and their classes are easy to be confused.

Our proposed method can be denoted by “MLR + MBD/MPD” in short, where MBD is the first recommended set-based dissimilarity, and MPD is the second recommended one. First, we demonstrate the effectiveness of the whole scheme in comparison with other representative methods. In this part, we evaluate the overall performances of the methods for all beat classes as well as their separate performances on each beat class. Second, we evaluate the components of the proposed model to justify their roles and test the model flexibility. In this part, we evaluate several different learned metric spaces for the recommended set-based dissimilarities, and compare these dissimilarities with other analogous ones. Moreover, we also evaluate the function of the regularizer of MLR as well as the minority size of MBD. Third, we discuss the critical parameters involved in the method to check how they influence the results. In this part, we pay attention to discussing the set size of MBD/MPD, as well as the trade-off parameter of MLR. Fourth, we validate the robustness of the proposed method by confirming its stability and reliability against different feature spaces. In this part, we evaluate the method by means of different features based on six wavelet types with two encoding ways. At last, we compare the proposed approach with other competitive techniques based on AAMI standards to show its compatibility and capability.

The evaluation rule is as follows. We perform 10-fold cross-validation. In each trial, we randomly halve the training and testing data. In addition, we keep the same train-test splits for method comparison. In the training stage, for the classes whose sizes are much larger than the others, such as classes N, A, L, R, P and V, we limit the training data number by randomly selecting at most 500 samples to alleviate the class imbalance problem. In the testing stage, we match between the testing and training sets to decide the testing set label. This process is on the basis of set-integrity assumption: the samples within the set are relevant.

There are three choices for instantiating the abstract operation of MBD in Equation (7). According to the preliminary tests on datasets MLII and MLV in Table 2, we find that the sum-operation is a little more effective than the other two, so we directly utilize the sum-operation. By the way, the choice of max-operation is also admissible for MBD because of its similar performance to the sum-operation.

### 4.2. Effectiveness Evaluation

We demonstrate the effectiveness of our proposed method in comparison with SVM [2], Neural Network (NN) [35], and Linear Discriminant Analysis (LDA) [4]. They belong to the traditional methods for ECG beat classification based on the single-sample manner. Hence, we treat single-sample classification in original feature space with Euclidean metric as the method baseline.

During implementation, for the proposed method, we set the testing set size to be 50 samples for each beat class except for types J, Q, e and S, because, in total, they only have 42 samples, 17 samples, eight samples, and one sample for testing, respectively. For classes J, Q, and e, we fix the set size to be five and, for S, we treat this single sample as the whole set. As for MBD, we set the minority size as the 1/10 sample number of the smaller one by default. If the minority size is smaller than one, we just maintain the size as one. For SVM, we select the Radial Basis Function (RBF) kernel with the default gamma value 2, set the penalty parameter to be 10, and make the stopping criterion as 0.01. For NN, we adopt the five-layer structure, in which the sizes from the bottom layer to the top layer are 36, 31, 26, 21 and 16, respectively. For the hidden units, we choose the hyperbolic tangent activation function. To learn such structure, we utilize the stochastic gradient descent algorithm, in which the batch size is set as half of the training data size, and the learning rate is set as two. For LDA, we keep the subspace dimension as 26 for dimension reduction.

The results on MLII and MLV are reported in Table 3 and Table 4, respectively, in terms of classification rate on Rank-1 and Rank-5. Here, the classification rate on Rank-1 is identical with accuracy, which is the ratio of the number of correctly classified patterns to the total number of patterns classified. For SVM and NN, we only record the accuracy because they directly produce the classification results rather than the distance scores for ranking. Note that, for multi-class classification, accuracy is more suitable than other evaluation metrics which are common in binary classification, such as sensitivity, specificity, and so forth. From the results, we can see that the proposed method significantly outperforms the compared widely-used approaches.

As our first recommended set-based dissimilarity, MBD performs better than MPD in the learned metric space. SVM and NN seem to drag down the performance because of the over-fitting and variation problems. However, these problems have been well handled by the proposed method by contrast. The advantage of our approach over the compared methods reflects the benefits from the suitably exploited set-based information in the learned metric space. To be frank, although tuning the parameters of these compared methods may perhaps bring some small performance fluctuation, such limited performance fluctuation will not influence the comparison results between our approach and them due to their significant performance gap.

We also evaluate the method performance for each independent beat class in Table 5 and Table 6. It can be observed from the results in Table 5 that, generally, the proposed method performs better than the other methods. For classes L, a, W, j, E, P and f, MLR + MBD even achieves saturated results. For classes e and S, our proposed method has unsatisfactory performance because the small sample number in their classes are adverse to exploiting the set-based information. However, for these two classes, the performances of other approaches are also quite low as well due to the limited training data. In addition, class Q displays an overall inferior performance for all methods as expected on account of its unclassifiable property. In Table 6, the proposed method outperforms other compared approaches on the whole. In addition, MLR + MBD acquires the saturated performance on classes L, a, j, E, P and f, which are similar to the results on MLII in Table 5. Moreover, class S fails all the methods. For e, although the proposed method is inferior to other methods, the winner also has quite low performance. Class Q is still unclassifiable as its label name suggests. Their low performances are also similar to the situation in MLII in Table 5.

### 4.3. Component Analysis

We also evaluate the two important modeling components in our method: metric learning and set-based dissimilarity.

On the one hand, we instantiate the metric learning component by several capable models other than MLR in the scheme. These models include Large Margin Nearest Neighbor (LMNN) [45], Information-Theoretic Metric Learning (ITML) [46], and Local Fisher Discriminant Analysis (LFDA) [47]. It can be seen from the results in Table 7 and Table 8 that, although the effectiveness of set-based dissimilarity MBD and MPD limits the performance enhancing space, MLR still behaves stronger than other compared metric learning models in boosting their performances. The performance gap between MLR + MBD/MPD and LMNN + MBD/MPD is not large. This is because MLR and LMNN share a similar inner mechanism despite their different modeling ways: they pursue an optimized relative comparison relationship between intra-class and inter-class distances. Therefore, LMNN can be used as an alternative metric learning component to substitute for MLR in the scheme. Such modeling freedom actually reflects the flexibility of our method, which also ensures its developing potentiality in the future.

On the other hand, we analyze the set-based dissimilarity component by comparing MBD, MPD, APD, and MAD [42]. They encode the point-to-point distances into the set level in different ways. MPD measures the minimum distance between sets, APD measures the average distance between sets, and MAD balances the strategies of MPD and APD by measuring the mean approach distance between sets, while MBD measures the minority distance between sets, which generalizes both MPD and MAD. The results in Table 7 and Table 8 show that MBD and MPD are substantially more effective than MAD and APD, which implies that the discriminative information is primarily contained within the outer part instead of the central part of the sets. Furthermore, the advantage of MBD over MPD indicates that, in the outer part of the sets, the inner minority is more discriminative than the outmost periphery.

Moreover, we also analyze the regularizer alternatives of the MLR component in Equation (5). The results in Table 9 and Table 10 show that changing the regularizer has a minor influence on the whole framework. This also embodies some flexibility of our method.

Furthermore, we evaluate the performance of MBD under different sizes of minorities formulated in Equation (7). The sizes of minorities are determined by $|A_{u}|$ and $|B_{v}|$. We use the same quantity of minorities for both measured sets: $|A_{u}\left|=\right|B_{v}\left|=\alpha =\beta \cdot \min(|A|,|B|\right)$, where *α* denotes the size of minorities and *β* denotes the ratio. If α<1, then we set α=1. The results for MBD in both Euclidean and learned metric spaces are reported in Table 11 and Table 12. We can see the effectiveness of minority-based strategy by comparing the results between using the partial subset from 1/50 to 1/2 and using the whole set by 1/1. The performance for both MBD and MLR + MBD present a rising tendency when *β* decreases by the large, respectively. In addition, when *β* is approximately within the range [1/30,1/6], MBD has relatively good performance in both Euclidean and learned metric spaces.

On the whole, MLR is a global metric learning method that optimizes the distance comparison relationship for all the samples in the feature space. Both effectiveness of MLR itself and improving the set-based dissimilarity for measurement based classification directly justify the goal of this model to make intra-class distances smaller than inter-class distances. In the learned metric space, local variations of the sample set become the main problem that impedes the performance of set-based dissimilarity. The effectiveness of MBD in such space just validates its capability to cope with such problem.

### 4.4. Parameter Discussion

We further discuss the critical parameters involved in the proposed method: the set size (the sample number of set) for MBD and the trade-off parameter for MLR. Because MPD is the second recommended set-based dissimilarity in our model, we also put it into discussion.

At first, we evaluate the performance of MBD and MPD in both Euclidean and learned metric spaces, respectively, under the set size varying from 10 to 100 with a step length of 10. The performance with the set size larger than 100 is less meaningful because the performance may be deteriorated or exaggerated due to the statistical bias caused by the limited sample number of a few beat classes.

By combining the results in Table 13 and Table 14, we find that the performance first increases and then decreases on the whole except the minor fluctuation. Hence, too large and too small set sizes may degrade the capability of both MBD and MPD; when the set size falls in the approximate range $\left[30,70\right]$, they can play comparably good performances in both Euclidean and learned metric spaces.

Then, we evaluate the MLR trade-off parameter *C* in Equation (5). This parameter plays the role in balancing slack variable and regularizer. Usually, when the different-class distributions are seriously overlapped in the feature space, the smaller parameter is more desirable, and, conversely, when the distributions are relatively separable, the larger one seems better. We testify a group of trade-off parameters {0.001,0.01,0.1,1,10,100,1000} to reveal how they influence the method performance of MLR + MBD/MPD. We also record the results of MLR under these trade-off parameters directly without doing set-based dissimilarity measurement. For convenience, we provide the baseline performance as reference.

By observing the results in Table 15 and Table 16, we find that, for MLR, the smaller parameters give rise to the better performance than the larger ones, which hints that the sample overlapping situation indeed exists in the feature space. Actually, the soft manner from the slack variable of MLR can cope with such a situation to a certain degree. In greater detail of the results, the comparison between the first and second rows shows the trade-off parameter has no influence on the superiority of MBD to MPD in the learned metric space; the comparison between the second and third rows manifests the effectiveness of set-based strategy in the learned metric space regardless of the settings of such parameters; the comparison between the third and fourth rows displays the positive role of metric learning itself in improving the space discriminability during single-sample classification.

### 4.5. Robustness Validation

The robustness of the proposed method can be demonstrated by its stable effective performance against different feature spaces as well as different datasets.

Firstly, we evaluate the proposed method in different feature space based on various wavelet transforms. These features are concatenated by either all or statistics (maximum, minimum, mean, and variance) of the decomposition coefficients of wavelets. The wavelets include Bior 6.8, Daubechies 14 (Db 14), Symlets 8, Coiflets 5, Fejer-Korovkin 22 (FK 22), and Reverse Bi-orthogonal 6.8 (RBior 6.8).

We can confirm the robustness of our approach by the results in Table 17 and Table 18 that the performances present a stable stair-wise increase from baseline through MLR + MPD to MLR + MBD irrespective of features and datasets. Moreover, the performance discrepancy on the same dataset is not that large among these features, which also shows some flexibility on feature component selection in the scheme. Furthermore, the features concatenated by all of coefficients seem to perform better than statistics of coefficients. This is because the latter is much higher compressed than the former, but the compressed representation is inevitably accompanied with the information loss. However, it is obvious that the full use of coefficients will lead to the high dimensionality of feature vectors, which will add to the burden of computing and storage. Actually, running methods based on features concatenated by statistics of coefficients takes much shorter time and smaller memory than those composed of all the coefficients. By contrast, the strategy using statistics of coefficients better regards the balance between effectiveness and efficiency of feature representation.

### 4.6. Technique Comparison

ECG beat classification is a well studied problem. In this paper, we deal with this issue from the 16-class classification perspective. This setting is different from much traditional research using AAMI standards (ANSI/AAMI EC57: 1998) that group ECG beats in the MIT-BIH Arrhythmia Database into five big classes [4,7,11,24,27,28]. To compare our method with these techniques in literature, we also conduct experiments on dataset MLII by means of such a widely-used standard classification scheme. Actually, the considered 16 classes contain all the 15 beat types addressed by the AAMI standards as well. According to the standards, the five big classes are Non-Ectopic Beat, Supra-Ventricular Ectopic Beat, Ventricular Ectopic Beat, Fusion Beat, and Unknown Beat. More specifically, Non-Ectopic Beat include beat types N, L, R, e and j; Supra-Ventricular Ectopic Beat include beat types a, A, S and J; Ventricular Ectopic Beat include beat types E and V; Fusion Beat include beat type F; and Unknown Beat include beat types Q, P and f.

In experiments, we use the feature composed of all decomposition coefficients of wavelet Db 14 due to the capable performance of this feature observed. We record the performance of the proposed method and the recent representative techniques in Table 19. This table shows good performance of the proposed method for ECG beat classification by the popular AAMI standards, which further confirms the method compatibility and capability as well.

## 5. Conclusions

In this paper, we proposed a novel method, set-based discriminative measure, to better resolve the issue of ECG beat classification. This method measures the discriminative set-based dissimilarity in a learned metric space. We have not only demonstrated the effectiveness, robustness, and flexibility of this method, but also analyzed its modeling components as well as critical parameters. Besides ECG beat classification, the proposed method also has potential to tackle other bio-information classification tasks that share similar characteristics to this issue. Therefore, our ongoing work in the future will include extending and applying this method to dealing with them.

## Figures and Tables

**Table 1:** Database properties.

Electrocardiogram (ECG) Beat Class	Class Size	Abbreviated Denotation
Normal Beat	75,023	N
Left Bundle Branch Block Beat	8072	L
Right Bundle Branch Block Beat	7255	R
Atrial Premature Beat	2546	A
Premature Ventricular Contraction	7129	V
Aberrated Atrial Premature Beat	150	a
Nodal (Junctional) Premature Beat	83	J
Supraventricular Premature or Ectopic Beat (Atrial or Nodal)	2	S
Fusion of Ventricular and Normal Beat	802	F
Ventricular Flutter Wave	472	W
Atrial Escape Beat	16	e
Nodal (Junctional) Escape Beat	229	j
Ventricular Escape Beat	106	E
Paced Beat	7026	P
Fusion of Paced and Normal Beat	982	f
Unclassifiable Beat	33	Q

**Table 2:** Comparison of different operations in Minority-Based Dissimilarity (MBD).

MBD	Sum-Operation	Max-Operation	Min-Operation
MLII	96.39	96.38	95.64
MLV	92.04	92.02	91.31

**Table 3:** Method performance for all beat classes on MLII.

Method	MLR + MBD	MLR + MPD	SVM	NN	LDA	Baseline
Rank-1	96.84	94.92	82.10	78.02	87.92	88.73
Rank-5	99.85	99.80	-	-	99.00	99.92

**Abbreviations**: Metric Learning to Rank→MLR; Minimum Point-wise Distance→MPD; Support Vector Machine→SVM; Neural Network→NN; Linear Discriminant Analysis→LDA.

**Table 4:** Method performance for all beat classes on MLV.

Method	MLR + MBD	MLR + MPD	SVM	NN	LDA	Baseline
Rank-1	92.78	91.10	74.49	76.38	83.89	83.49
Rank-5	99.74	99.69	-	-	99.01	98.21

**Table 5:** Method performance for each beat class on MLII.

Beat	MLR + MBD	MLR + MPD	SVM	NN	LDA	Baseline
N	96.72	94.72	81.05	74.36	85.26	86.38
L	100	99.75	91.94	90.63	98.33	98.37
R	99.03	99.03	93.84	89.60	96.20	95.32
A	94.40	90.40	76.58	68.19	82.82	86.00
V	96.90	92.54	98.43	78.82	87.10	87.24
a	100	100	53.60	35.60	67.60	67.60
J	81.25	81.25	70.71	22.62	80.95	76.19
S	0.00	0.00	0.00	0.00	10.00	50.00
F	98.75	82.50	67.73	78.35	87.53	87.43
W	100	85.00	36.48	79.92	83.73	85.97
e	10.00	10.00	25.00	6.25	36.25	38.75
j	100	95.00	77.91	61.83	81.48	81.22
E	100	100	88.30	86.23	93.77	93.96
P	100	100	61.28	94.49	98.71	98.91
f	100	100	82.24	87.07	96.62	97.19
Q	16.67	16.67	2.35	10.00	20.59	6.47

**Table 6:** Method performance for each beat class on MLV.

Beat	MLR + MBD	MLR + MPD	SVM	NN	LDA	Baseline
N	91.91	90.11	70.36	73.89	80.52	80.56
L	100	100	89.49	91.46	97.44	96.46
R	95.97	97.22	89.85	89.61	93.26	92.97
A	86.40	81.60	77.71	62.22	79.46	81.00
V	94.23	89.15	82.44	57.41	80.22	76.15
a	100	100	40.40	13.87	59.47	56.67
J	80.00	80.00	68.57	42.38	72.14	76.19
S	0.00	0.00	0.00	0.00	0.00	0.00
F	77.50	71.25	43.22	74.16	85.26	83.04
W	80.00	75.00	42.37	77.58	82.80	73.77
e	0.00	0.00	12.50	1.25	21.25	18.75
j	100	95.00	80.78	69.22	77.74	77.48
E	100	100	76.42	81.89	89.81	89.81
P	100	100	84.66	96.90	99.37	98.87
f	100	100	83.60	90.57	96.90	95.15
Q	3.33	3.00	0.00	2.94	20.59	2.35

**Table 7:** Modeling component analysis on MLII.

**Modeling**	**MLR + MBD**	**LMNN + MBD**	**ITML + MBD**	**LFDA + MBD**	**MLR + MPD**	**LMNN + MPD**
Accuracy	96.84	96.65	96.39	95.87	94.92	94.61
**Modeling**	**ITML + MPD**	**LFDA + MPD**	**MBD**	**MPD**	**MAD**	**APD**
Accuracy	94.19	93.70	96.39	94.19	30.68	4.46

**Abbreviations**: Large Margin Nearest Neighbor→LMNN; Information-Theoretic Metric Learning→ITML; Local Fisher Discriminant Analysis→LFDA; Mean Approach Distance→MAD; Average Point-wise Distance→APD.

**Table 8:** Modeling component analysis on MLV.

** Modeling**	** MLR + MBD**	** LMNN + MBD**	** ITML + MBD**	** LFDA + MBD**	** MLR + MPD**	** LMNN + MPD**
Accuracy	92.78	92.43	92.04	91.37	91.10	90.57
**Modeling**	**ITML + MPD**	**LFDA + MPD**	**MBD**	**MPD**	**MAD**	**APD**
Accuracy	90.00	90.04	92.04	90.00	16.22	4.30

**Table 9:** MLR regularizer analysis on MLII.

Regularizer	MLR + MBD	MLR + MPD	MLR
tr(w)	96.84	94.92	89.42
${∥w∥}^{2}/2$	96.81	94.93	89.41

**Table 10:** MLR regularizer analysis on MLV.

Regularizer	MLR + MBD	MLR + MPD	MLR
tr(w)	92.78	91.10	84.79
${∥w∥}^{2}/2$	92.96	90.96	85.42

**Table 11:** Minority size analysis for MBD on MLII.

**Size Ratio**	**1/1**	**1/2**	**1/3**	**1/4**	**1/5**	**1/6**	**1/7**
MLR + MBD	86.46	93.19	94.95	95.89	96.57	96.64	96.66
MBD	85.34	93.54	94.99	95.80	96.28	96.47	96.43
**Size Ratio**	**1/8**	**1/9**	**1/10**	**1/20**	**1/30**	**1/40**	**1/50**
MLR + MBD	96.84	96.84	96.84	96.25	95.90	94.92	94.92
MBD	96.50	96.50	96.39	95.67	95.29	94.19	94.19

**Table 12:** Minority size analysis for MBD on MLV.

**Size Ratio**	**1/1**	**1/2**	**1/3**	**1/4**	**1/5**	**1/6**	**1/7**
MLR + MBD	85.66	90.38	90.90	91.37	92.10	92.31	92.69
MBD	80.77	90.42	90.79	91.03	91.54	91.56	91.69
**Size Ratio**	**1/8**	**1/9**	**1/10**	**1/20**	**1/30**	**1/40**	**1/50**
MLR + MBD	92.87	92.87	92.78	92.95	92.66	91.10	91.10
MBD	91.80	91.80	92.04	92.08	91.75	90.00	90.00

**Table 13:** Set size discussion for set-based dissimilarity on MLII.

Set Size	10	20	30	40	50	60	70	80	90	100
MLR + MBD	93.84	95.77	96.31	96.76	96.84	96.79	96.64	96.37	96.37	95.83
MLR + MPD	93.84	94.47	94.79	94.78	94.92	94.96	94.67	94.60	94.64	94.28
MBD	93.40	95.34	95.96	96.15	96.39	96.34	96.36	96.02	96.01	95.62
MPD	93.40	93.98	94.26	94.31	94.19	94.51	94.13	94.14	94.23	93.90

**Table 14:** Set size discussion for set-based dissimilarity on MLV.

Set Size	10	20	30	40	50	60	70	80	90	100
MLR + MBD	90.05	92.27	92.89	92.79	92.78	92.75	92.41	92.28	92.15	91.95
MLR + MPD	90.05	90.64	90.86	91.04	91.10	91.08	90.82	90.69	90.76	90.43
MBD	89.41	91.18	91.91	92.04	92.04	91.91	91.54	91.10	91.37	91.00
MPD	89.41	89.69	89.93	89.85	90.00	90.08	90.03	89.70	89.77	89.64

**Table 15:** MLR trade-off parameter discussion on MLII.

Trade-Off Parameter	0.001	0.01	0.1	1	10	100	1000
MLR + MBD	96.83	96.76	96.80	96.84	96.79	96.82	96.82
MLR + MPD	94.92	94.94	94.89	94.92	94.87	94.86	94.86
MLR	89.45	89.45	89.41	89.42	89.41	89.43	89.44
Baseline	88.73	88.73	88.73	88.73	88.73	88.73	88.73

**Table 16:** MLR trade-off parameter discussion on MLV.

Trade-Off Parameter	0.001	0.01	0.1	1	10	100	1000
MLR + MBD	92.96	92.96	92.96	92.78	92.87	93.01	92.69
MLR + MPD	90.96	90.96	90.96	91.10	91.16	91.17	90.70
MLR	85.42	85.42	85.42	84.79	83.71	83.98	83.72
Baseline	83.49	83.49	83.49	83.49	83.49	83.49	83.49

**Table 17:** Method performance in different feature spaces on MLII.

**All of Coefficients**	**Bior 6.8**	**Db 14**	**Symlets 8**	**Coiflets 5**	**FK 22**	**RBior 6.8**
MLR + MBD	96.93	97.83	97.68	97.64	97.83	97.55
MLR + MPD	94.39	96.26	95.93	95.87	96.01	95.76
Baseline	92.18	92.54	92.37	92.39	92.49	92.16
**Statistics of Coefficients**	**Bior 6.8**	**Db 14**	**Symlets 8**	**Coiflets 5**	**FK 22**	**RBior 6.8**
MLR + MBD	96.84	93.95	96.48	96.17	94.28	96.56
MLR + MPD	94.92	92.31	94.31	93.99	92.69	94.76
Baseline	88.73	82.78	83.34	83.81	83.87	84.05

**Abbreviations**: Bi-orthogonal 6.8→Bior 6.8; Daubechies 14→Db 14; Fejer-Korovkin 22→FK 22; Reverse Bi-orthogonal 6.8→RBior 6.8.

**Table 18:** Method performance in different feature spaces on MLV.

**All of Coefficients**	**Bior 6.8**	**Db 14**	**Symlets 8**	**Coiflets 5**	**FK 22**	**RBior 6.8**
MLR + MBD	94.40	94.20	94.44	93.94	94.18	94.27
MLR + MPD	92.57	92.31	92.83	92.67	92.60	92.50
Baseline	89.57	89.64	89.80	89.50	89.61	89.60
**Statistics of Coefficients**	**Bior 6.8**	**Db 14**	**Symlets 8**	**Coiflets 5**	**FK 22**	**RBior 6.8**
MLR + MBD	92.78	92.43	93.06	93.00	93.11	92.89
MLR + MPD	92.04	90.51	91.62	91.14	90.68	91.30
Baseline	83.49	81.43	83.23	82.61	82.06	83.25

**Table 19:** Performance comparison of ECG classification techniques.

Literature	Representation	Classification	Accuracy
Martis et al. [4]	DWT + ICA	Probabilistic NN	99.28
Elhaja et al. [7]	PCA + DWT + HOS + ICA	SVM-RBF	98.91
Martis et al. [28]	PCA	SVM-RBF	98.11
Das et al. [11]	ST + DWT + TF	Multilayer Perceptron NN	97.50
Chazal et al. [27]	Morphology + Intervals	LDA	96.87
Thomas et al. [24]	DTCWT + MF	Artificial NN	94.64
Proposed	DWT	MLR + MBD	99.36

**Abbreviations**: Discrete Wavelet Transform→DWT; Independent Component Analysis→ICA; Principal Component Analysis→PCA; Higher Order Spectra→HOS; S-Transform→ST; Temporal Features→TF; Dual Tree Complex Wavelet Transform→DTCWT; Morphological Features→MF.
